# Inequality in the distribution of health resources and health services in China: hospitals versus primary care institutions

**DOI:** 10.1186/s12939-017-0543-9

**Published:** 2017-03-03

**Authors:** Tao Zhang, Yongjian Xu, Jianping Ren, Liqi Sun, Chaojie Liu

**Affiliations:** 1 School of Medicine, Hangzhou Normal University, Hangzhou, Zhejiang 310036 China; 2 School of Public Policy and Administration, Xi’an Jiaotong University, Xi’an, Shaanxi 710000 China; 3 School of Psychology and Public Health, La Trobe University, Melbourne, 3086 Australia

**Keywords:** Equity, Hospitals, Primary care institutions, Health resources, Health services

## Abstract

**Background:**

Equity is one of the major goals of China’s recent health system reform. This study aimed to evaluate the equality of the distribution of health resources and health services between hospitals and primary care institutions.

**Methods:**

Data of this study were drawn from the China Health Statistical Year Books. We calculated Gini coefficients based on population size and geographic size, respectively, for the indicators: number of institutions, number of health workers and number of beds; and the concentration index (CI) for the indicators: per capita outpatient visits and annual hospitalization rates.

**Results:**

The Gini coefficients against population size ranged between 0.17 and 0.44 in the hospital sector, indicating a relatively good equality. The primary care sector showed a slightly higher level of Gini coefficients (around 0.45) in the number of health workers. However, inequality was evident in the geographic distribution of health resources. The Gini coefficients exceeded 0.7 in the geographic distribution of institutions, health workers and beds in both the hospital and the primary care sectors, indicating high levels of inequality. The CI values of hospital inpatient care and outpatient visits to primary care institutions were small (ranging from -0.02 to 0.02), indicating good wealth-related equality. The CI values of outpatient visits to hospitals ranged from 0.16 to 0.21, indicating a concentration of services towards the richer populations. By contrast, the CI values of inpatient care in primary care institutions ranged from -0.24 to -0.22, indicating a concentration of services towards the poorer populations. The eastern developed region also had a high internal inequality compared with the other less developed regions.

**Conclusion:**

Significant inequality in the geographic distribution of health resources is evident, despite a more equitable per capita distribution of resources. Richer people are more likely to use well-resourced hospitals for outpatient care. By contrast, poorer people are more likely to use poorly-resourced primary care institutions for inpatient care. There is a risk of the emergence of a two-tiered health care delivery system.

## Background

Despite great progress in health system reform, inequity in medical resources and medical care services remains a serious concern of many researchers and policymakers in China. Empirical evidence suggests that large inequity in resources and services can exacerbate disparities in health outcomes and quality of life [1, 2]. In recent years, the Chinese government has endorsed equity as an important indicator for health system evaluation [3, 4]. With almost universal coverage of social health insurance, health care accessibility and affordability of the poor and disadvantaged populations emerged to be top of the governmental agenda for further policy development [5].

In China, hospitals and primary care institutions deliver the majority of medical care services although, public health agencies and other institutions also play a role [6]. Hospitals are able to attract more and quality resources (such as a health workforce and health technologies) than their primary care counterparts. However, due to the large amount of primary care institutions and better accessibility, primary care can play a more crucial role in promoting regional health equity [7]. In comparison with hospitals, unfortunately, a serious shortage of resources exists in primary care institutions in China, leading to distrust from consumers. Services delivered by primary care institutions are deemed poor quality. They are unable to fulfill a gatekeeping function in the health system. It is common for consumers to seek medical attention from hospitals for minor illness or chronic conditions. Driven by the market forces, more resources have been poured into hospitals, further exacerbating disparities between hospitals and primary care institutions [8–10].

Recently, inequity in medical resources and medical care services started to attract attention from researchers: some focused on certain medical conditions [11–13]; others explored inequity in resources and services within the primary care sector [14, 15]. Hung argued that a big gap in resources and services between hospitals and primary care institutions exists and it continues to be increase despite the government’s favorable investment policies for the primary care sector [16]. But there is paucity in the literature documenting inequity in resources and services between hospitals and primary care institutions.

This study aimed to evaluate equity in medical resources and medical care services between hospitals and primary care institutions in China.

## Methods

### Data source

Data used in this study were extracted from the China Statistical Yearbook and the China Health Statistics Yearbook from 2010 to 2014, which covered 31 provinces and autonomous regions municipalities. Due to inconsistencies of data standards, Macao, Hong Kong and Taiwan were excluded from the study.

The 31 provinces were divided into three economic zones according to their geographical location and Gross Domestic Product (GDP) per capita: western undeveloped zone, central developing zone and eastern developed zone. The eastern developed zone covered Beijing, Tianjin, Hebei, Liaoning, Shandong, Jiangsu, Zhejiang, Shanghai, Fujian, Guangdong and Hainan. The central developing zone covered Heilongjiang, Jilin, Shanxi, Henan, Anhui, Hubei, Jiangxi and Hunan. The western undeveloped zone covered Inner Mongolia, Ningxia, Gansu, Shaanxi, Sichuan, Chongqing, Guizhou, Yunnan, Guangxi, Qinghai, Xinjiang and Tibet [17].

In the statistics yearbooks, hospitals were identified by their registration certificates. These includes general hospitals, TCM (Traditional Chinese Medicine) hospitals, hospitals of integrated medicine, and specialized hospitals. There were three levels of hospitals: primary, secondary and tertiary. Most hospitals were owned by governments (public hospitals). Primary care institutions reported in the statistics included urban community health centers & stations, rural health centers, village clinics, and independent outpatient clinics. They were smaller in size, but many still had beds and could admit patients with non-urgent conditions [17]. We did not include public health agencies, maternal and child health care centers, and nursing homes in this study, although they also delivered a small percentage of clinical medical care.

### Measurements of inequity

The Gini coefficient and concentration index (CI) have been identified as superior tools for measuring inequity [18]. The Gini coefficient examines the distribution of health resources and services against population size and geographic size, while the CI assesses the distribution of health resources and services against economic status [19]. The Gini coefficient was calculated based on the Lorenz curve: a graphical representation of a function of the cumulative proportion of resources or services of ordered institutions mapped onto the corresponding cumulative proportion of their size. It reflects the ratio of the area between the Lorenz curve and the diagonal line, to the whole area below the 45° line.

$$ {S}_1=1/2{\displaystyle \sum_{i=0}^{i=1}\left({Y}_i+{Y}_{i+1}\right){X}_{i+1}} $$

$$ \mathrm{G}=2\left(0.5\hbox{-} {\mathrm{S}}_1\right) $$

Where S_1_ is the area bounded by the Lorenz curve and the axes; Y_i_ is the cumulative proportion of health resources or services (Y_0_ = 0); and X_i+1_ is the cumulative proportion of each group of the population or geographical area. The G ranges from 0 to 1; a value of 0 indicates equitable distribution of resources or services; a value of less than 0.3 shows preferred equity status; a value of greater than. 0.4 triggers an alert of inequity; a value exceeding 0.6 reflects a highly inequitable state [20].

The concentration index defined as twice the area between the concentration curve (cumulative proportion of resources/services mapped onto the corresponding cumulative proportion of wealth) and the line of equality: C = 2cov(x, h)/μ.

Where x is fractional rank in terms of per capita GDP; h is the health (resource or service) indicator; and μ is the mean of the health indicator. The range of C lies in between -1 and 1: a value of zero indicates absolute equity; a negative value indicates a concentration of health resources or services on the poorer populations; a positive value represents a concentration of health resources or services on the richer populations [21]. Due to limitations of data availability, we did not use standardization in estimating CI.

### Indicators

We chose two groups of indicators for measuring inequity in line with previous studies [22, 23], reflecting the distributions of health resources and health services, respectively. Health resources were measured by number of institutions, number of beds and number of health workers. Health services utilization was measured by average outpatient visits per person and the annual hospitalization rate in the served communities [24].

## Results

### Equity in the distribution of health resources

From 2010 to 2014, health resources increased in both hospitals (Table 1) and primary care (Table 2) sectors in terms of average resources per capita or per km^2^. The increasing trend was observed in all of the three economic zones. Hospital resources appeared to rise a more rapid pace than primary care resources. Overall, there were more primary care institutions than hospitals. However, most health workers and beds were allocated to hospitals.

**Table 1:** Distribution of health resources in the hospital sector (2010–2014)

Year	Economic zone	Population (1000 persons)	Geographic area (1000 km^2^)	Hospitals		Health workers		Beds	
				Per 1000 persons	Per 1000 km^2^	Per 1000 persons	Per 1000 km^2^	Per 1000 persons	Per 1000 km^2^
2010	Total	1333850	9610.300	0.018	7.030	3.397	1815.158	2.675	1308.351
	Eastern	50035	96.518	0.017	14.536	4.042	4141.334	2.879	2880.044
	Central	52845	208.738	0.017	4.363	3.128	850.486	2.540	693.699
	Western	30058	573.225	0.021	1.929	2.985	325.945	2.577	277.400
2011	Total	1340420	9610.300	0.019	7.306	3.587	1905.550	2.886	1387.176
	Eastern	50405	96.518	0.017	15.030	4.240	4327.138	3.042	3018.988
	Central	52968	208.738	0.018	4.597	3.273	899.447	2.744	759.821
	Western	30185	573.225	0.021	2.033	3.198	356.495	2.837	309.585
2012	Total	1347890	9610.300	0.020	7.638	3.831	2043.209	3.165	1504.222
	Eastern	50773	96.518	0.018	15.696	4.945	4611.838	3.290	3230.241
	Central	53139	208.738	0.018	4.742	3.499	976.448	3.042	853.326
	Western	30357	573.225	0.022	2.183	3.442	399.808	3.132	355.967
2013	Total	1355160	9610.300	0.020	8.071	4.122	2178.967	3.466	1623.555
	Eastern	51098	96.518	0.018	16.474	4.744	4890.802	3.507	3445.684
	Central	53339	208.738	0.019	4.953	3.708	1050.623	3.332	945.499
	Western	30531	573.225	0.024	2.448	3.827	445.347	3.518	405.307
2014	Total	1362460	9610.300	0.021	8.416	4.370	2313.813	3.723	1731.190
	Eastern	51418	96.518	0.019	17.175	4.980	5173.344	3.713	3642.380
	Central	53559.000	208.738	0.019	5.116	3.921	1122.929	3.616	1033.950
	Western	30699.000	573.225	0.025	2.588	4.111	486.499	3.803	444.091

**Table 2:** Distribution of health resources in the primary care sector (2010 to 2014)

Year	Economic zone	Population (1000 persons)	Geographic area (1000 km^2^)	Primary care institutions		Health workers		Beds	
				Per 1000 persons	Per 1000 km^2^	Per 1000 persons	Per 1000 km^2^	Per 1000 persons	Per 1000 km^2^
2010	Total	1333850	9610.300	0.698	207.315	2.381	979.316	0.846	335.204
	Eastern	50035	96.518	0.546	343.328	2.282	1902.253	0.692	611.752
	Central	52845	208.738	0.707	205.396	2.539	758.200	0.930	281.703
	Western	30058	573.225	0.832	83.916	2.367	280.701	0.931	117.369
2011	Total	1340420	9610.300	0.723	209.758	2.496	1012.334	0.868	338.192
	Eastern	50405	96.518	0.545	345.298	2.320	1973.743	0.696	607.258
	Central	52968	208.738	0.721	209.765	2.562	769.625	0.956	290.618
	Western	30185	573.225	0.888	85.509	2.613	292.848	0.969	123.264
2012	Total	1347890	9610.300	0.720	209.793	2.537	1036.512	0.922	351.748
	Eastern	50773	96.518	0.545	348.568	2.362	2031.398	0.716	614.744
	Central	53139	208.738	0.705	203.854	2.557	771.048	1.031	315.257
	Western	30357	573.225	0.890	86.543	2.684	301.510	1.038	134.995
2013	Total	1355160	9610.300	0.716	210.990	2.585	1067.879	0.940	356.560
	Eastern	51098	96.518	0.540	351.431	2.424	2107.795	0.714	616.445
	Central	53339	208.738	0.701	204.025	2.559	774.800	1.034	318.930
	Western	30531	573.225	0.886	86.895	2.750	310.007	1.085	143.418
2014	Total	1362460	9610.300	0.713	212.187	2.604	1085.242	0.958	360.404
	Eastern	51418	96.518	0.539	354.795	2.422	2146.990	0.713	613.118
	Central	53559	208.738	0.698	203.922	2.562	778.634	1.065	330.572
	Western	30699	573.225	0.883	86.972	2.798	316.378	1.112	148.638

Large regional disparities appeared in the number of resources per km^2^, despite small regional differences in the number of resources per capita. The eastern developed region had a much higher level of density in the distribution of hospitals, health workers and beds. For example, in 2014, the number of hospitals per 1000 km^2^ in the eastern zone was 8 times more than that in the western zone. Similarly, in 2014, the number of primary care institutions per 1000 km^2^ in the eastern zone was 4 times more than that in the western zone.

The Gini coefficients against population size ranged between 0.17 and 0.44 in the hospital sector: 0.36–0.44 for the number of hospitals, 0.23–0.28 for the number of health workers, and 0.17–0.26 for the number of beds respectively, indicating relatively good equality (Table 3). The primary care sector showed a slightly higher level of Gini coefficients (around 0.45) in the number of health workers. But the distribution of primary health care institutions and beds was equitable, with Gini coefficients ranging from 0.02 to 0.27 (Table 3).

**Table 3:** Gini coefficients of population and geographic distribution of health resources (2010–2014)

Gini coefficient	Year	Hospital sector			Primary care sector		
		Institutions	Health workers	Beds	Institutions	Health workers	Beds
Population size	2010	0.44	0.28	0.26	0.23	0.43	0.04
	2011	0.41	0.26	0.24	0.27	0.43	0.04
	2012	0.36	0.24	0.18	0.27	0.45	0.02
	2013	0.37	0.23	0.18	0.27	0.45	0.07
	2014	0.36	0.23	0.17	0.26	0.45	0.07
Geographic size	2010	0.86	0.90	0.88	0.77	0.81	0.82
	2011	0.86	0.89	0.87	0.78	0.81	0.81
	2012	0.83	0.89	0.83	0.78	0.83	0.81
	2013	0.81	0.89	0.82	0.78	0.84	0.81
	2014	0.81	0.88	0.82	0.89	0.88	0.81

However, inequality was evident in the geographic distribution of health resources (Table 3). The Gini coefficients exceeded 0.7 in the geographic distributions of institutions, health workers and beds in both the hospital and the primary care sectors, indicating high levels of inequality. No obvious changes in Gini coefficients were found over the years from 2010 to 2014.

### Equity in utilization of health services

Primary care institutions provided more outpatient services (2.508–2.938 visits per person a year) than hospitals (1.634–2.283 visits per person a year); whereas, hospitals provided more inpatient services (7.183%–11.178% admission rates) than primary care institutions (2.619%–2.637% admission rates).

From 2010 to 2014, the most significant increase in the utilization of health services occurred in inpatient services in the hospital sector, compared with a slight increase in outpatient services in both sectors and inpatient services in the primary care sector (Table 4).

**Table 4:** Health services utilization in hospital and primary care sectors (2010–2014)

Year	Economic zone	Per capita GDP(Yuan)	Hospitals		Primary care institutions	
			Outpatient visits (times)	Inpatient care (%)	Outpatient visits (times)	Inpatient care (%)
2010	Total	28736.935	1.634	7.183	2.508	2.619
	Eastern	44670.364	2.443	7.624	2.862	1.680
	Central	21001.125	1.091	6.701	2.252	2.982
	Western	19288.500	1.259	7.100	2.355	3.238
2011	Total	39136.161	1.793	8.018	2.621	2.496
	Eastern	56799.909	2.693	8.401	2.995	1.560
	Central	30759.250	1.176	7.573	2.387	2.861
	Western	28529.000	1.379	7.963	2.433	3.110
2012	Total	43351.032	1.978	9.346	2.789	2.789
	Eastern	61907.091	2.969	9.517	3.199	1.633
	Central	34264.625	1.317	8.930	2.558	3.237
	Western	32398.917	1.509	9.467	2.567	3.549
2013	Total	47046.581	2.123	10.261	2.919	2.814
	Eastern	66765.364	3.158	10.214	3.386	1.565
	Central	37064.750	1.406	9.781	2.660	3.238
	Western	35625.583	1.653	10.623	2.664	3.676
2014	Total	50734.742	2.283	11.178	2.938	2.637
	Eastern	71753.909	3.371	11.050	3.459	1.490
	Central	39753.000	1.532	10.741	2.698	3.104
	Western	38788.333	1.786	11.586	2.619	3.376

Higher levels of health services utilization in the eastern developed zone (except for inpatient services in primary care institutions) was found compared with those in the central developing and western undeveloped zones. Western residents were more likely to use primary care institutions for inpatient care than their eastern and central counterparts (Table 4).

The CI values of hospital inpatient care and outpatient visits to primary care institutions were small (ranging from -0.02 to 0.02), indicating good wealth-related equality (Table 5). The CI values of outpatient visits to hospitals, ranged from 0.16 to 0.21, indicated a concentration of services towards the richer populations. By contrast, the CI values of inpatient care in primary care institutions ranged from -0.24 to -0.22, indicating a concentration of services towards the poorer populations.

**Table 5:** Concentration index (CI) of health services in the hospital and primary care sectors (2010–2014)

Year	Hospitals		Primary care institutions	
	Outpatient visits	Inpatient care	Outpatient visits	Inpatient care
2010	0.20	-0.01	-0.01	-0.24
2011	0.21	0.02	0.02	-0.23
2012	0.16	0.02	-0.02	-0.23
2013	0.21	-0.01	0.02	-0.22
2014	0.20	-0.05	0.02	-0.24

**Fig. 1:**
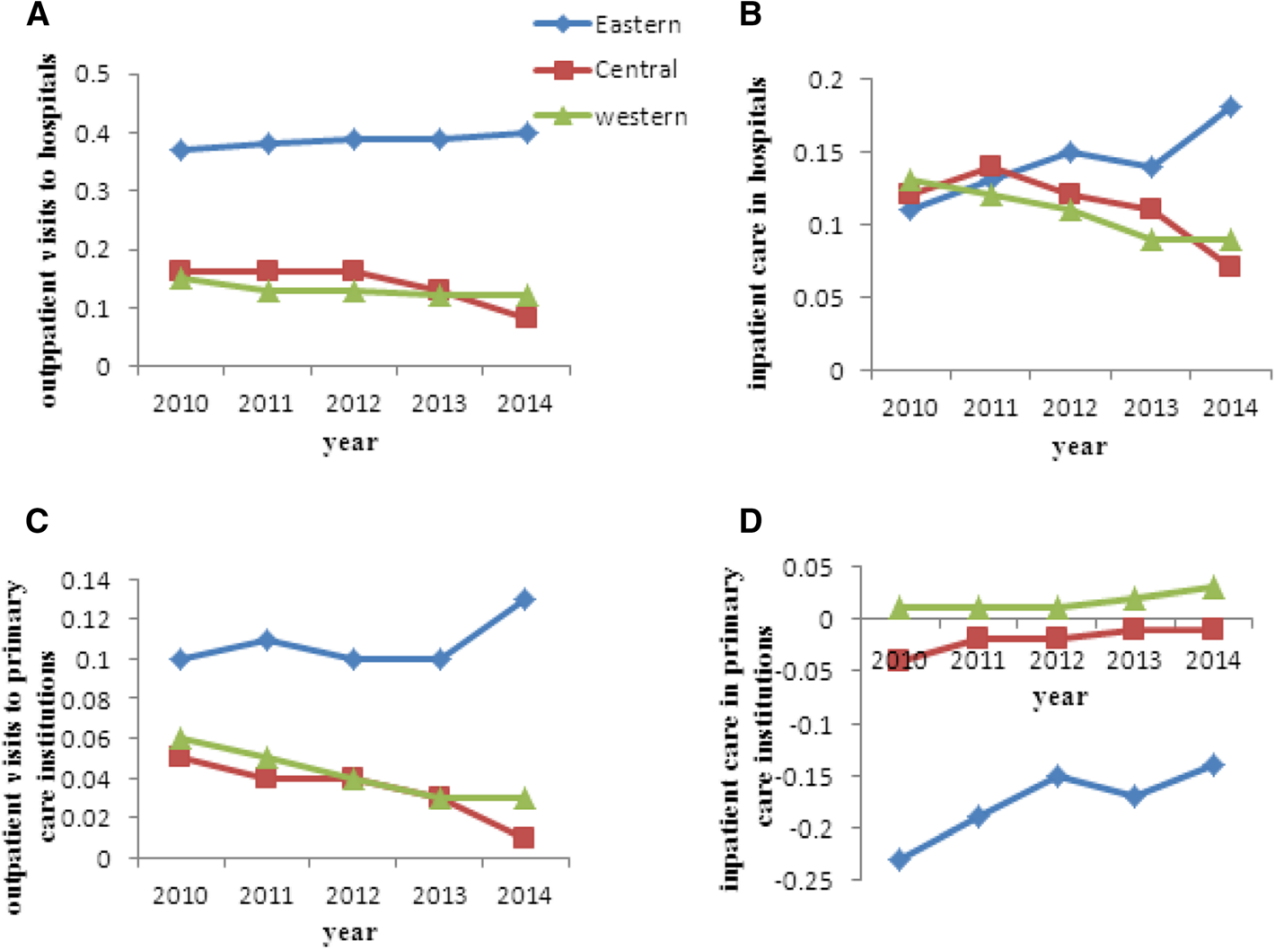
Concentration index (CI) of health services utilization in the eastern, central and western regions from 2010 to 2014. **a** and **b** illustrate changes in CI for outpatient visits to and inpatient care in hospitals, respectively; **c** and **d** illustrate changes in CI for outpatient visits to and inpatient care in primary care institutions, respectively. The X-axis represents the year and the Y-axis represents the value of CI

Within each economic zone, wealth-related inequality in health services also existed (Fig. 1). The eastern developed zone had a higher level of CI than the central developing and western undeveloped zones, indicating greater inequality. In the eastern developed zone, poorer people were more likely to use primary care institutions for their inpatient care, while the other services were favored by richer people. From 2010 to 2014, the CI in the central and western zones declined, compared with an increasing trend in the eastern zone, except for inpatient care in primary care institutions.

## Discussion

This study compared the hospital and primary care sector in China in terms of the distributions of health resources and health services. We found that most health workers and beds were allocated to hospitals despite there being a much larger number of primary care institutions (in China, primary care institutions comprise more than 96% of all health institutions [6]). Meanwhile, hospitals developed more rapidly than primary care institutions. This may impose a risk of further enlarging the capacity gap between hospitals and primary care institutions, jeopardizing the governmental effort to strengthen primary care. The most recent round of health reform launched in 2009 has a strong focus on primary care. Although some progress has been made with increased governmental investments in primary care, the capacity development of primary care still lags behind the hospital sector. Several other studies in China also found that quality resources tend to be increasingly concentrated in hospitals [13, 25]. Low wages and lack of career opportunities have often been blamed for the shortage of primary care workers and the loss of quality health workers to hospitals [6].

Inequality in health resources is mainly reflected in geographic distributions (density) instead of per capita (population) distribution. The Gini coefficients exceeded 0.7 in the geographic distribution of resources, much higher than those of per capita population distribution of resources. This is not surprising given that most resource planning programs have considered population size [26]. But few have been concerned about the geographic density of resources. Conversely, health services operated in scarcely populated, large geographic catchments are often poorly resourced because they are more expensive to operate [27]. However, residents living in less populated areas, such as rural districts, are often disadvantaged in China with lower income and less support. Those people deserve more policy attention.

We found significant regional disparities in the distribution, in particular the geographic distribution of health resources. The eastern developed region has a higher level of resources than the other two regions. This finding is consistent with other studies [27]. The larger size and the higher density of populations in the eastern region mean that its operational costs of health services are relatively cheaper. Meanwhile, the decentralized governmental budgeting process means that the wealthy eastern provinces have a higher financial capacity to fund health services. The health services in the eastern region can also offer a better salary and welfare to health workers, enticing quality health workers from the less developed regions to move to the east. Indeed, previous studies show that.most quality health resources are concentrated in hospitals, especially in tertiary hospitals, of which 46.68% are located in the eastern provinces in China. It was estimated that 30% of provinces in China have attracted 50% of quality health resources [6].

The outpatient visits to primary care institutions outnumbered those to hospitals, not only because the number of primary care institutions is large, but also because they are geographically close to residents, easy to access, and cheap. Understandably, most people chose hospitals for inpatient care. What is concerning is that the volume of inpatient care grew much faster than outpatient care over the years from 2010 to 2014, in particular for those provided by the hospital sector. This is aligned with the stronger growth of hospital resources. At the same time, the growth of outpatient care services was marginal, indicating a relatively weaker primary care sector.

Regional disparities exist in health services utilization. Residents living in the eastern developed zone were more likely to use hospitals for outpatient care than their poorer central and western counterparts. On the other hand, residents living in the western undeveloped zone were more likely to use primary care institutions for inpatient care than their eastern and central counterparts. This is clearly associated with the gap in income level and health expenditure [28–30]. The CI indicates that outpatient care provided by hospitals tends to concentrate on the richer; whereas, inpatient care provided by primary care institutions tends to concentrate on the poorer. Such a two-tier system is worrisome. Empirical evidence shows that both the overuse of services (such as hospital care) and the under use of services (such as primary care) coexist in China [31].

The internal disparities within each economic zone further illustrate the regional differences in the equality of health care. The eastern developed region has a much higher level of inequality compared with the other regions. Similar results were also found in previous studies [32]. The inequality in outpatient care and hospital inpatient care, as indicated by the CI values, was actually growing in the eastern region, enlarging the regional gaps in CI. In addition, the regional CI gap in inpatient care provided by primary care institutions, the only service that tends to concentrate on the poor, shrank over the years. The less developed regions have to learn how to mitigate the risk of growing inequality in their development process.

This study has several policy implications. Firstly, more health resources, especially quality health worker, should be allocated to primary care institutions in order to narrow the capacity gap between hospitals and primary care institutions. Secondly, regional disparities need to be addressed. This can only be done through financial transfer coordinated by the central government. The current governmental budgeting system and the social health insurance arrangements in China are highly decentralized and fragmented, preventing the central government from fulfilling this role. Thirdly, more attention needs to be paid to the potential emergence of a two-tier system, where the well-resourced facilities (such as hospitals) favor the rich and the poorly resourced facilities (such as primary care institutions) favor the poor. Finally, a tiered health care delivery system needs to be developed, in which the function of primary care institutions complements that of hospitals. Consumers should be able to access different services based on their health care needs, not their ability to pay.

### Limitations

This study analyzed a 5-year trend in the change of equality of health resources and health services. It would be interesting to perform further analyses on the longer term changes when data are made available. The indicators selected in this study were restricted by the availability of data. Although they are consistent with other studies, they may not be comprehensive enough to reflect the entire picture of inequality in health resources and health services. For example, no significant inequality in hospital inpatient care was observed in this study. However, the less affluent patients are more likely to end their hospital stay prematurely than their more affluent counterparts due to financial barriers imposed by deductible and co-payment requirements. Unfortunately, those indictors are not available at this stage. We are also unable to decompose CI due to the limited availability of data.

## Conclusion

Significant inequality in the geographic distribution of health resources is evident, despite a more equitable per capita distribution of resources. The residents living in the eastern developed region are more likely to use the well-resourced hospitals for outpatient care. By contrast, the residents living in the western undeveloped region are more likely to use the poorly-resourced primary care institutions for inpatient care. Apart from regional disparities, inequality within each region also exists. The wealth-related inequality in the eastern developed region is increasing, further enlarging the regional gaps in CI.
